# Identifying the active microbes driving organosulfur cycling from taurine and methionine in marine sediment

**DOI:** 10.1093/ismeco/ycaf033

**Published:** 2025-02-25

**Authors:** Ömer K Coskun, William D Orsi, Steven D’Hondt, Gonzalo V Gomez-Saez

**Affiliations:** Department of Earth and Environmental Sciences, Ludwig-Maximilians-Universität München, Richard-Wagner Str. 10, 80333 Munich, Germany; Department of Earth and Environmental Sciences, Ludwig-Maximilians-Universität München, Richard-Wagner Str. 10, 80333 Munich, Germany; GeoBio-Center^LMU^, Ludwig-Maximilians-Universität München, Richard-Wagner Str. 10, 80333 Munich, Germany; Graduate School of Oceanography, University of Rhode Island, 215 S Ferry Rd, Narragansett, 02882 Rhode Island, United States; Department of Earth and Environmental Sciences, Ludwig-Maximilians-Universität München, Richard-Wagner Str. 10, 80333 Munich, Germany; GeoBio-Center^LMU^, Ludwig-Maximilians-Universität München, Richard-Wagner Str. 10, 80333 Munich, Germany

**Keywords:** marine sediment, carbon cycle, dissolved organic sulfur, taurine, methionine, qSIP, Gammaproteobacteria, Deltaproteobacteria, *Neptuniibacter*, *Methanococcoides*

## Abstract

Studies on microbial sulfur cycling in marine sediment have primarily centered on the cycling of inorganic sulfur. The microbial diversity underlying the cycling of organosulfur compounds is largely unexplored. In this study, we present the first quantification of dissolved organic sulfur (DOS) microbial assimilation in marine surface sediments using ^13^C-DOS quantitative DNA stable isotope probing (qSIP). We sampled marine sediment from 493 m water depth on the Puerto Rico continental slope, measured ^13^C-assimilation from two DOS substrates (^13^C-taurine and ^13^C-methionine), and compared the ^13^C-DOS assimilation to ^13^C-glucose uptake. Taurine utilization was confined to bacteria, whereas methionine was degraded by bacteria and archaea, including methanogenic *Methanococcoides*. Globally widespread uncultivated clades of Gammaproteobacteria and Deltaproteobacteria were the main drivers of DOS cycling and exhibited increased assimilation of carbon from taurine and methionine, compared to glucose. Only one operational taxonomic unit (OTU) affiliated with *Neptuniibacter* was found to assimilate taurine and methionine, but not glucose, implying that microbes exclusively utilizing both DOS substrates as a carbon source in marine sediments are rare. Still, a substantial number of bacterial taxa exhibited a higher assimilation of ^13^C from taurine or methionine, compared to glucose, indicating their preference for both DOS substrates over glucose as a carbon source in the sediment. These results represent the first quantitative assessment of organosulfur cycling from taurine and methionine by uncultivated microbes in a marine benthic environment.

## Introduction

Marine dissolved organic sulfur (DOS), as part of dissolved organic matter (DOM), represents the largest reservoir of organic sulfur in the ocean (>6.7 Pg S; [1]). The DOS pool consists of a myriad of compounds with concentrations in the nanomolar range and varying sulfur redox states ranging from −2 to +6 [2, 3]. Recent studies have identified an abundant and diverse suite of marine bacteria with the genetic capacity for DOS transformation [2]. Dimethylsulfoniopropionate (DMSP) stands out as the most extensively studied DOS substrate, primarily due to its conversion into dimethylsulfide (DMS), a volatile DOS compound with a crucial role in regulating Earth’s climate in the atmosphere [4]. Marine phytoplankton transform methionine into DMSP [5], which makes up half of the sulfur within their cell biomass [6]. Bacterioplankton have also been observed incorporating reduced sulfur from DMSP back into methionine [7], highlighting the vital role of interconversions among DOS compounds in ecosystem functioning. Besides, methionine plays an essential role as the initiator of the protein translation [8], a key component of the cofactor S-adenosylmethionine [9] and a precursor in the formation of cysteine and taurine [10]. Methionine concentration in the ocean can range between 0.2–0.69 nM and its turnover time can be faster than some sugars such as glucose, suggesting that exogenous amino acids might be an important preferred carbon, nitrogen and/or sulfur source for microbial communities [11]. Taurine is a non-proteinogenic amino sulfonic acid, which is widely synthesized by many organisms including marine metazoans [12] and algae [13, 14]. Dissolved taurine concentrations vary depending on the environment. In coastal environments, it can reach up to 30 nM [15] while in the epipelagic and bathypelagic waters taurine concentrations range between 0.2–16 nM and 0.07–1.6 nM, respectively [16]. Diverse marine prokaryotes exhibit metabolic capabilities to use taurine either as an energy or a carbon source [17]. Furthermore, taurine has also been recently highlighted as a key intermediate for host- symbiont interaction in tropical sponges, emphasizing the important role of these biogenic organosulfur compounds in ecosystem functioning [18]. However, there is a lack of information regarding the diversity of marine microbes that mediate turnover of methionine and taurine.

Previous research on microbial sulfur cycle in the sediment mainly focused on the cycling of inorganic sulfur. Only a limited number of studies have investigated the biotransformations of organosulfur compounds in anoxic marine sediments [19–22]. However, the microbial diversity involved in the cycling of organosulfur compounds remains largely unexplored [23, 24]. After pyrite, organosulfur compounds are the second largest reservoir of sulfur in sediments, with at least 35% of the total sulfur compounds being sulfonates [25, 26]. Microbial metabolism plays an important role in the production, degradation, and recycling of DOS in marine sediments, especially in continental shelves and slopes [27]. In marine sediments, DOS is primarily produced by microbial assimilatory sulfate reduction (ASR) [23] and indirectly through dissimilatory sulfate reduction (DSR) [24]. Sulfate reduction is quantitatively the most abundant and relevant anaerobic respiration process in global marine sediments [24, 28, 29]. However, many prokaryotes may also fulfill their sulfur requirements from reduction of elemental sulfur (S^0^) or different polysulfides that act as sulfur intermediates [30]. Furthermore, the flux of organosulfur molecules from the surface waters to the sediments via sinking particulate organic sulfur may represent another important source of DOS into the benthos [31]. Global climate change will likely lead to an increase in certain DOS compounds like taurine over the next few decades [3] which might impact the benthic microbial communities [32]. Altogether, it is clear that microbial communities in marine sediments are key players in the formation and uptake of DOS compounds. However, the diversity of microbes that utilize DOS compounds as a carbon source in marine sediments is not well understood.

One of the major challenges in microbial ecology is to link the identities of microorganisms to the roles they play in nature. DNA stable isotope probing (DNA-SIP) offers a solution that uses isotopically labeled elements in various substrates to link elemental fluxes physically to an organism’s genome [33, 34]. Moreover, quantitative DNA-SIP (qSIP) measures the amount of substrate assimilated by individual microbial populations (e.g. ^13^C, ^15^N, ^18^O) [35]. Here, we used qSIP to provide novel quantitative measurements on DOS utilization by specific microbial groups in marine sediments. We applied DOS substrates of varying oxidation states (^13^C-taurine and ^13^C-methionine) and compared this to ^13^C-glucose assimilation. The qSIP experiments were complemented with gas chromatography quadrupole mass spectrometry (GC-QMS) measurements to quantify the ^13^CO_2_-remineralization of ^13^C-substrate added by the benthic microbial communities.

## Materials and methods

### Sampling sediments at Puerto Rico Trench

**Figure 1:**
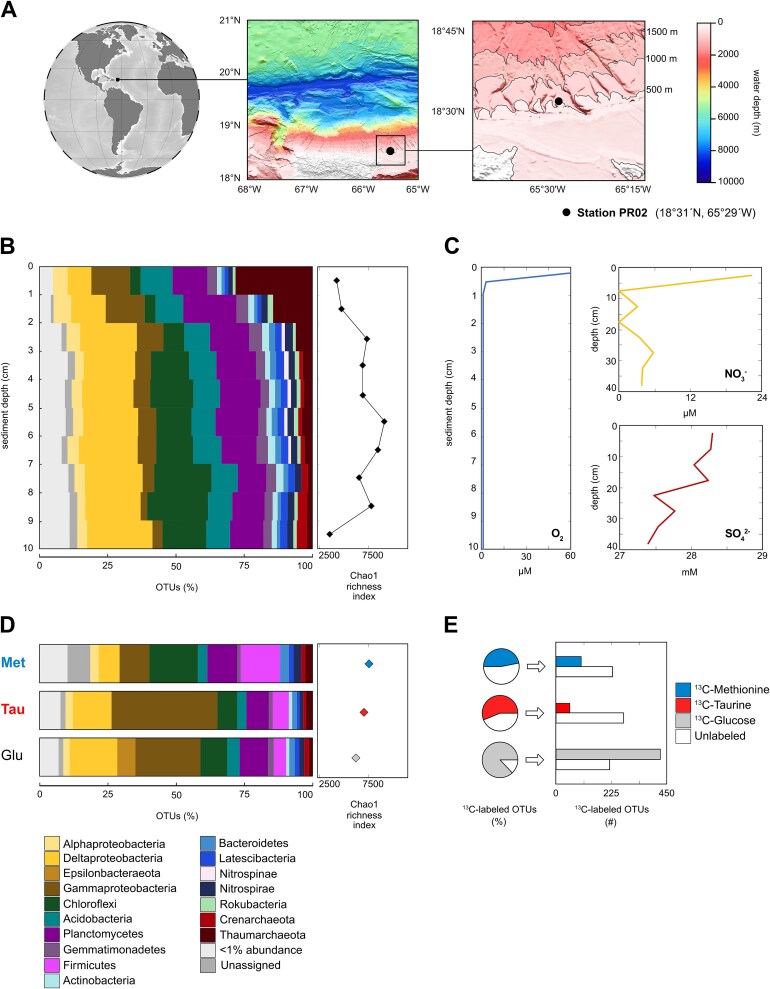
**Study area, geochemistry and microbial community composition**. (A) Location of the study area (black dot) and bathymetry of Puerto Rico Trench. (B) Vertical profile of *in situ* microbial diversity of 16S rRNA genes and Chao1 richness index. (C) Geochemical porewater profiles of O_2_, NO_3_^−^, and SO_4_^2−^. (D) Microbial diversity of 16S rRNA genes at the end (10-days) of the three different qSIP incubations and Chao1 richness index. (E) Relative abundance of ^13^C-labeled OTUs after 10-days of incubation and corresponding number of OTUs that were determined to be ^13^C-labeled from each qSIP incubation.

Puerto Rico Trench sediment samples were obtained during the RV Neil Armstrong expedition AR64-02 on February 2022. A 50 cm long sediment core was collected using a multi-corer (diameter 10 cm) from 493 m water depth on the Puerto Rico continental slope (Station PR02; 18°31′N; 65°29′W; Fig. 1A). CTD profiles showed that the *in situ* water temperature at 493 m was 13°C. Upon the retrieval, the temperature of the sediment in the cores was measured as 19°C and incubations were performed at 16°C. Porewater subsamples using Rhizon samplers were obtained for geochemical analyses at 2.5, 7.5, 12.5, 17.5, 22.5, 27.5, 32.5, and 38 cm sediments depths and stored at −20°C for further processing. Afterwards, the sediment cores were sectioned every cm (0–1, 1–2, 2–3, 3–4, 4–5, 5–6, 6–7, 7–8, 8–9, and 9–10 cm) within the same day. Untreated sediment samples (t_0_) of 0.5 g to assess the initial microbial community composition were immediately stored at −20°C for further processing in the laboratory.

### Experimental setup

For qSIP incubations, 1.5 g sediment from the top 2 cm of the core were placed into 2 ml glass vials (Carl Roth GmbH, Karlsruhe, Germany) using a sterile spatula, and the vials were filled to the top with 0.5 ml sterile artificial seawater (30 mM MgCl_2_·6H_2_O, 16 mM MgSO_4_·7H_2_O, 2 mM NaCO_3_, 10 mM KCl, 9 mM CaCl_2_, 450 mM NaCl) leaving no air in the headspace. Sediment slurries in each vial were incubated for either 30-h or 10-days in the dark with one of the following 500 μg / g of ^13^C-labeled substrates: methionine (335 nM; 16.8 μmol ^13^C per g), taurine (400 nM; 8 μmol ^13^C per g), and glucose (277 nM; 16.7 μmol ^13^C per g). As control experiments, we prepared incubations with the ^12^C carbon sources added at the same concentration (e.g. [33, 35]). As we could not measure the natural concentration of these substrates *in situ* and there is a lack of literature regarding the concentration of DOS substrates in the Puerto Rico marine sediments, we made the following assumptions based on the existing literature: the total dissolved free amino acids in sediments from Norwegian-Greenland Sea ranged between 1–11 μM [36] and percentage of each taurine and methionine to total amino acids in Lowe Cove US sediments was <2% [37]. Thus, we estimated taurine and methionine concentrations between 20–220 nM in marine sediments. Although the added DOS substrate concentrations were higher than natural levels (e.g. taurine <30 nM in coastal settings [15]), they remained lower than the recommended substrate concentration of ∼50 μmol ^13^C per g sediment, which is necessary for sufficient DNA-SIP detection of active microorganisms [38]. Thus, we acknowledge that our experimental setup might potentially create perturbations in the microbial activity, however in return, sufficient ^13^C-labeling was ensured for qSIP analysis without exceeding the recommended concentrations [35, 38]. Dissolved oxygen (O_2_) concentrations were measured throughout the incubations using sterile non-invasive fiber optic oxygen sensor spots (PreSens, Germany) which were fixed to the inside wall of the glass vials before adding the sediments (Fig. S1). After the termination of the different incubation time points, incubation flasks were stored at −20°C and shipped to the laboratory at LMU Munich (Germany).

### Gas chromatography quadrupole mass spectrometry (GC-QMS): Respired ^13^CO_2_ measurements

To quantify the remineralization of ^13^C-substrate added to the SIP incubations, we determined the relative amount of ^13^CO_2_ produced using GC-QMS. Specifically, 0.2 g of incubation material was added to 20-ml gas tight glass vials that were crimp sealed, heated to 60°C for 5 min and 1 ml of headspace gas was sampled via a headspace auto sampler connected to a gas chromatograph with a quadrupole mass spectrometer as the detector (GC–MS-QP2020 NX, Shimadzu, Japan). N_2_ was used as the carrier gas. The GC–QMS was calibrated for trace gas analysis with a pre-separation column (U-Bond, 0.32 mm ID, 10 μm film, 30 m) and a second column (Carboxen-1010 Plot, 0.32 mm ID, 15 μm film, 30 m) for chromatographic separation of trace gases. The elution time for CO_2_ (6.2 min) was determined by comparing to a CO_2_ standard (99.99%, Linde Gas, Germany). The relative amount of remineralized carbon from the added labeled substrates was calculated as percentage of ^13^CO_2_ (*m/z* = 45) relative to the naturally abundant ^12^CO_2_ (*m/z* = 44), released from the sample matrix into the flask headspace. Detection limit of 1% was determined after consideration of the natural abundance of ^13^C in the unlabeled samples.

### Quantitative DNA stable isotope probing (qSIP): DNA extraction, density gradient centrifugation and fractionation, qPCR and metagenomics

**Figure 2:**
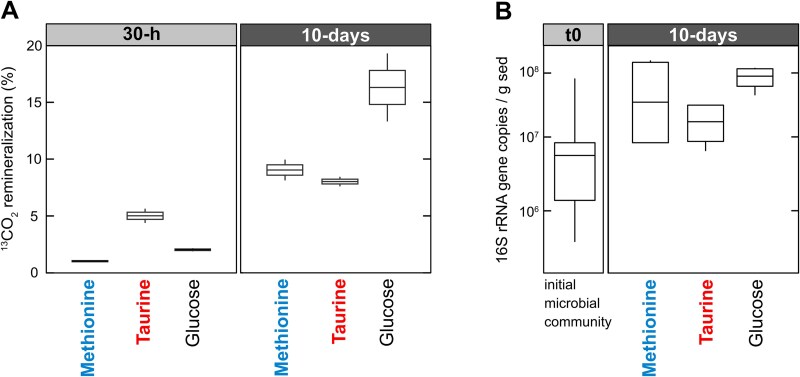
^**13**^ **CO**_**2**_ **-remineralization from DOS mirrors microbial abundance**. (A) ^13^CO_2_ remineralization from DOS substrates (taurine, methionine) and glucose at 30-h and 10-days. (B) Concentration of 16S rRNA gene copy numbers in the initial samples (t_0_) and after the termination of the qSIP incubations at 10 days.

DNA was extracted from initial samples (t_0_) and SIP experiments following standard procedure (e.g. [39]). ^13^C-enrichment in the 30-h incubations was below the minimum threshold required for qSIP (Fig. 2A) [35]. Thus, we applied qSIP only to the 10-days incubations. Ten-fold diluted DNA samples were prepared for density gradient centrifugation according to previously defined protocols for qSIP [40]. For each SIP experiment, we conducted three individual ultracentrifugation runs which served as technical replicates. Although biological replicates are more ideal in SIP incubations, it is important to note that the technical variation in excess ^13^C-excess atom fraction (EAF) resulting from ultracentrifugation and density fractionation is often comparable to or could even exceed the variation in biological ^13^C-EAF between incubations in replicate tubes [39–41].

Quantitative polymerase chain reaction (qPCR) to evaluate the DNA abundance in the density fractions of each incubation was performed using universal primers (515F-Y/806RB [42, 43]) targeting the V4 hypervariable region of 16S ribosomal ribonucleic acid (rRNA) genes. qPCR reactions were carried out in a CFX Connect real-time PCR (Bio Rad, Hercules, CA, USA) as described previously [39]. Two 16S rRNA gene PCR amplicons from each density fraction (technical replicates to minimize PCR bias) were pooled and subjected to dual-indexed barcoded sequencing of 16S rRNA gene amplicons on the Illumina MiniSeq (Illumina, San Diego, CA, USA) at LMU Munich (Germany) following an established protocol [44]. Quality trimming and assembling of the MiniSeq reads, contaminants removal, and removal of low abundance taxa from density gradient fractions were done following the established protocol in [45]. The raw operational taxonomical units (OTUs) table consisted of 21848 OTUs. After contamination removals, 17005 OTUs were used for downstream analysis. Following the further selection criteria [45], both datasets for taurine and methionine incubations included 332 OTUs while glucose dataset had 642 OTUs.

The ^13^C-EAF values of OTUs were calculated according to a previously described study [35] using the HTSSIP R package [46] (Data S1). For the statistical analyses, non-metric multidimensional scaling (NMDS) and plots, phyloseq [47] and vegan packages (https://github.com/vegandevs/vegan) were used in R.Studio Version 3.3.0 [48]. EAF measurements of ^13^C-labeled OTUs in each experiment with varying ^13^C-substrates enabled us to test whether the utilization of these compounds was significantly associated with specific phylogenetic groups (e.g. “phylogenetic organization” [49]). 16S rRNA genes of these ^13^C-labeled OTUs were aligned with MUSCLE [50] embedded in SeaView [51]. The resulting fasta file was given to W-IQ-TREE [52] with an option to select the best phylogenetic model using Bayesian criterion, which resulted in SYM + I + G4 algorithm using ModelFinder [53]. The Blomberg’s K measurements were calculated in phytools (https://github.com/liamrevell/phytools) with 1000 iterations using ^13^C-labeling of OTUs (i.e. EAF measurements) and the constructed phylogenetic tree from W-IQ-TREE. Each experiment was run separately using the same phylogenetic tree as an input. For phylogenetic analysis of ^13^C-labeled Asgard archaea, we constructed phylogenetic tree by using the closely-related 16S rRNA genes together with the OTUs identified in a comparable qSIP study [54]. The same methodological steps were also applied for the construction of this phylogenetic tree and the K2P + G4 algorithm was chosen using ModelFinder [53].

Metagenomes were produced from the heavy fractions of ^13^C-labeled density gradients (Fig. S2; gray shaded area) and unfractionated DNA from ^13^C-labeled SIP experiments using Nextera XT DNA Library Prep Kit (Illumina, San Diego, CA, USA) following [45]. SqueezeMeta [55] was used for downstream analysis using co-assembly mode with default settings. Metagenomic dataset and intermediate files were deposited under https://figshare.com/s/84f617866cbd2324e35d. The 16S rRNA amplicon sequences, metagenomic sequence data were entered in the NCBI Sequence Read Archive under BioProject ID PRJNA1136705.

## Results

### Geochemistry

The sediment core contained >60 μM of O_2_ in the top cm but became anoxic (<1 μM) within a few cm below the seafloor (cmbsf) (Fig. 1C). The nitrate (${\textrm NO}_{3}^{-}$) concentration was 22 μM at the core top, but decreased to a few μM in 8 cmbsf. Sulfate concentrations decreased gradually from 28.5 mM at the core top to 27.5 mM at 25 cmbsf, presumably due to microbial sulfate reduction, indicating a continuous flux of sulfate to greater depths (Fig. 1C). Surface sediments were supplied to the qSIP incubations, which included ^13^C-glucose, ^13^C-taurine, and ^13^C-methionine. In all incubations, O_2_ was rapidly consumed to levels below detection within the first 5-h, resulting in anoxic conditions for the remaining 10-days (Fig. S1). By the end of the incubation period, we observed black precipitates in many of the incubation vials (Fig. S1), indicating iron-sulfide mineral crystallization that is suggestive of increased sulfate-reducing microorganisms (SRM) activity [29]. After 30-h, remineralized ^13^CO_2_ was <2% in the incubations receiving ^13^C-glucose and ^13^C-methionine, while the ^13^C-taurine incubation exhibited remineralized ^13^CO_2_ concentrations of 5.1 ± 0.8% (average ± standard deviation of the percentage of total CO_2_-released; Fig. 2A). This suggests that taurine remineralization was faster than methionine or glucose. After 10-days of incubation, the experiments with ^13^C-glucose, ^13^C-taurine and ^13^C-methionine exhibited higher ^13^CO_2_ remineralization rates. The ^13^C-glucose incubations had approximately double ^13^CO_2_ remineralization on average (16.7 ± 4.0%) compared to ^13^C-taurine and ^13^C-methionine incubations (8.1 ± 0.6% and 9.1 ± 1.2%, respectively) (Fig. 2A).

### Microbial community composition

The microbial community composition (16S rRNA gene) of the top 3 cmbsf was dominated by OTUs affiliated with Thaumarchaeota and Gammaproteobacteria (Thaumarchaeota: 28 to 2%; Gammaproteobacteria: 14 to 2%; Fig. 1B). Below 3 cmbsf, Deltaproteobacteria and Chloroflexi relative abundance increased with depth (Deltaproteobacteria: 8 to 24%; Chloroflexi: 4 to 22%; Fig. 1B). After 10-days of incubation, the microbial community composition was different depending on the added substrates (Fig. 1D). Incubations with glucose were dominated by Gammaproteobacteria (24%), Deltaproteobacteria (17%), Planctomycetes (10%), and Chloroflexi (10%). Taurine incubations showed a large increase in Gammaproteobacteria (39%), followed by Deltaproteobacteria (14%), Planctomycetes (8%), and Chloroflexi (7%). The most dominant phyla detected in methionine incubations were Chloroflexi (18%), Firmicutes (14%), and Planctomycetes (11%). In comparison to the glucose and taurine incubations, the proportion of Gammaproteobacteria and Deltaproteobacteria in the methionine incubations were considerably lower with 11 and 8% of total relative abundance, respectively (Fig. 1D). NMDS analysis and microbial richness index (Chao1) showed that microbial composition after 10-days of incubation were more closely clustered with those of 2–8 cm sediment (anoxic) than those of 0–1 cm sediment layer (Fig. 1B, S3).

### Microbial abundance and overall ^13^C-incorporation

In our experiments, we observed a consistent pattern between the 16S rRNA gene copy numbers and ^13^C-remineralization rates after 10-days (Fig. 2). The initial concentration of 16S rRNA genes was 1.4 ± 2.5 (average ± standard deviation) x 10^7^ copies per g of sediment (Fig. 2B). By the end of the incubation period, the ^13^C-glucose, ^13^C-methionine, and ^13^C-taurine incubations showed a substantial increase in 16S rRNA gene copies compared to the initial conditions (Fig. 2B). In order to quantify the assimilation of ^13^C into microbial DNA, we measured the shift in DNA buoyant density between ^12^C and ^13^C-labeled treatments [35] resulting from the ^13^C-glucose and ^13^C-DOS substrates (Supplementary Fig. S2). This showed that incubations amended with ^13^C-glucose, ^13^C-methionine, and ^13^C-taurine all had a visible increase in DNA buoyant density compared to the corresponding ^12^C-incubations (Supplementary Fig. S2).

### ^13^C-labeling of operational taxonomic units

**Figure 3:**
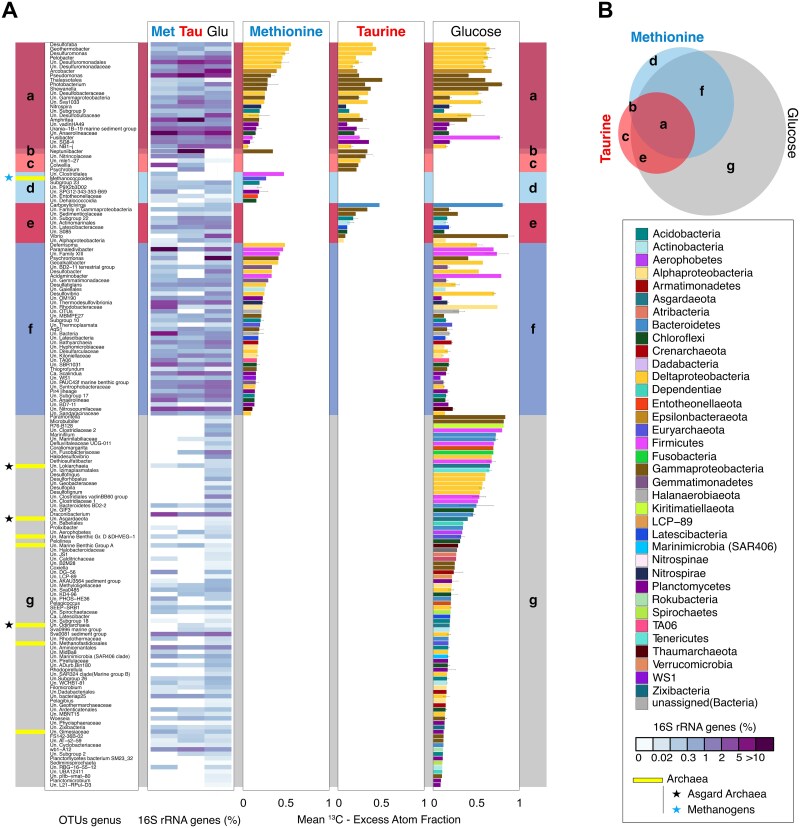
**Quantitative labeling of carbon assimilating taxa and their overlap between DOS and glucose incubations**. (A) Relative abundance (heatmap) and EAF values (histograms) of carbon assimilating taxa in the qSIP incubations. The left-hand side bars represent groups of shared, or not shared, ^13^C-labeled taxa between incubations (see Venn diagram, panel B). The error bars display the standard deviation of the mean. Histograms are colored by group. (B) Venn diagram shows the number of overlapping ^13^C-labeled OTUs between the three qSIP incubations. The unique letter assigned to each area corresponds to groupings, which were used as the overlapping categories on the left side of panel A.

The degree of ^13^C-labeling within OTUs was quantified by measuring ^13^C-EAF in 16S rRNA gene of each detectable OTU in the sample. EAFs were calculated on a scale of zero to one, where one corresponds to 100% incorporation of ^13^C into the DNA fragments containing 16S rRNA genes (Figs 3 and S4, Data S1). After 10-days of incubation, the proportion of OTUs that were determined to be ^13^C-labeled was 17% in ^13^C-taurine (57 OTUs), 31% in ^13^C-methionine (103 OTUs), and 66% in ^13^C-glucose (424 OTUs; Figs 1E and S4). The relative abundance of these ^13^C-labeled OTUs was 47, 56, and 87% for ^13^C-methionine (103 OTUs), ^13^C-taurine (57 OTUs), and ^13^C-glucose (424 OTUs) incubations, respectively (Fig. 1E). The mean EAF values of ^13^C-labeled OTUs in ^13^C-taurine and ^13^C-methionine incubations were nearly identical (0.22 ± 0.10; 0.23 ± 0.13; mean ± standard deviation, respectively). In contrast, ^13^C-labeled OTUs in the ^13^C-glucose incubations exhibited the highest mean EAF (0.27 ± 0.2 EAFs) (Fig. S4). These findings suggest that taurine and methionine utilization were limited to a smaller number of OTUs compared to glucose, and that in general DOS was assimilated to a lesser extent than glucose as a carbon source.

**Figure 4:**
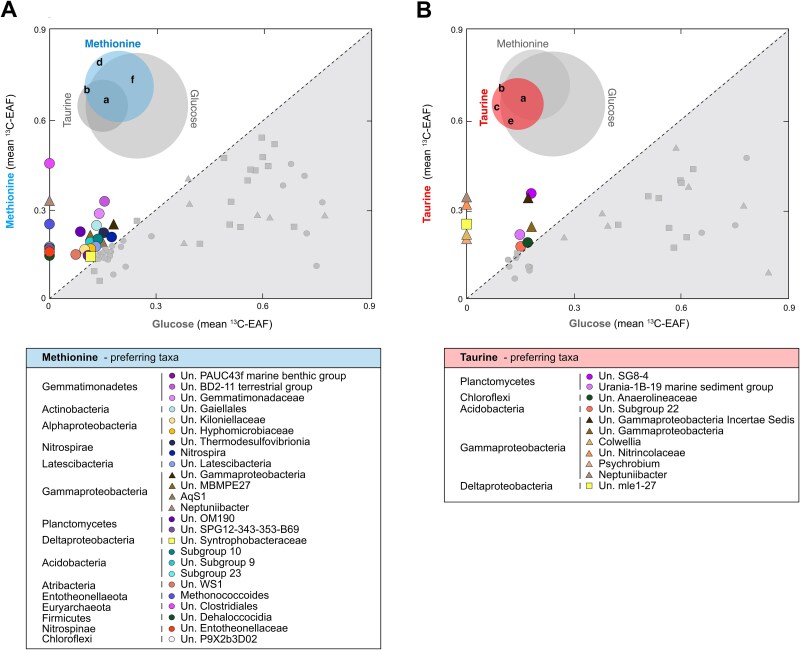
**Uncultivated bacterial taxa exhibiting a preference for DOS**. ^13^C-EAF values of OTUs that assimilated methionine (A) or taurine (B) were plotted against the corresponding ^13^C-EAF from ^13^C-glucose. OTUs that plot above the dotted 1:1 line are interpreted as exhibiting a preference for these DOS substrates. The Venn diagrams display the OTUs that were selected for the plot (see Fig. 3). For genera with more than one OTU, each point represents the mean EAFs calculated from the average of bootstrapped median EAF (Fig. S4) for the corresponding OTUs. ^13^C-labeled OTUs not assigned to a genus-level taxon were represented as “unassigned” (Un.) together with the most representative taxonomic information. Colored symbols (above the dotted 1:1 line) represent OTUs that had increased ^13^C-EAF from the DOS substrates taurine, or methionine, relative to glucose.

**Figure 5:**
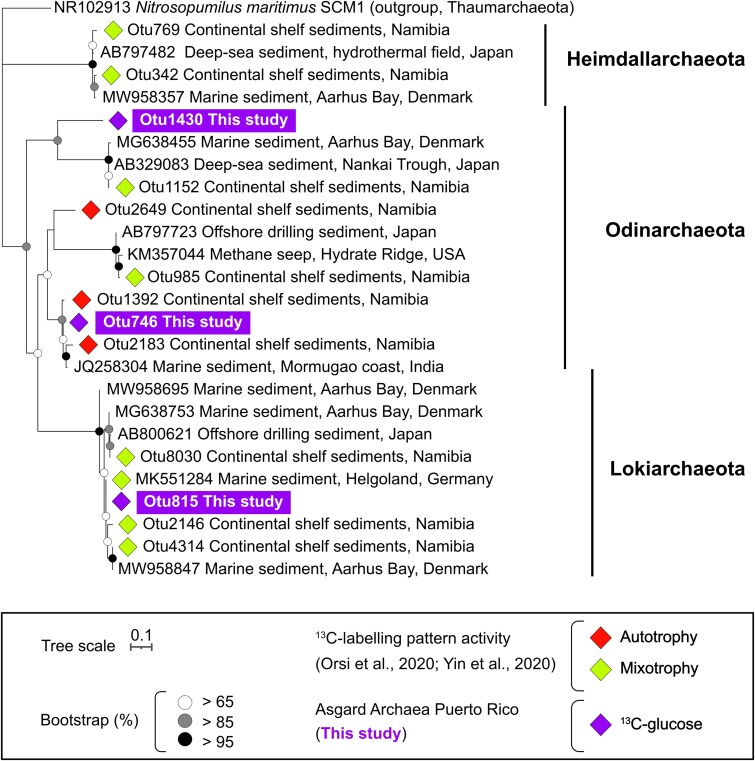
**Asgard archaea assimilating**  ^**13**^**C-glucose**. The tree is based on 16S rRNA gene V4 hypervariable regions, and includes three OTUs from our study (boxes) that displayed significant ^13^C-glucose assimilation (diamonds). The metabolic preferences of OTUs (mixotrophy and autotrophy) detected in other SIP studies [54, 56] are included in the tree to compare them with OTUs from this study.

^13^C-labeled OTUs were grouped into 168 genera (Figs 3, 4, and S4), half of which utilized ^13^C-DOS together or independently of glucose (“a–f”; 84 OTUs; Fig. 3), and half of which were exclusive to the glucose incubation (“g” = 84; Fig. 3). ^13^C-labeled OTUs utilizing ^13^C-methionine, ^13^C-taurine, and ^13^C-glucose corresponded to 24 distinct genera, comprising 14, 34, and 37% of total microbial communities, respectively (“a” = 24; Fig. 3). Two thirds of them belonged to Deltaproteobacteria and Gammaproteobacteria (Fig. 3). One genus that assimilated DOS substrates, which did not assimilate^13^C-glucose (“b” = 1; Figs 3 and 4) was a ^13^C-labeled OTU (0.33 EAF) affiliated with *Neptuniibacter* which represented 21% and 0.3% relative abundances in taurine and methionine incubations, respectively. Exclusive ^13^C-taurine assimilating OTUs were affiliated with Proteobacteria genera (“c” = 4; Figs 3 and 4). In contrast, the exclusive ^13^C-methionine assimilating microbes were much more diverse, and spanned 7 different phyla (“d” = 7; Figs 3 and 4). The highest degree of ^13^C-glucose utilization was detected for OTUs affiliated with *Vibrio* (2 OTUs; 0.85 ± 0.07 EAF), *Paramoritella* (1 OTU; 0.82 EAF), and *Microbulbifer* (1 OTU; 0.80 EAF). In addition, Asgard archaeal OTUs belonging to unassigned (Un.) genera under class Lokiarchaeia (1 OTU; 0.65 EAF), class Odinarchaeia (1 OTU; 0.19 EAF) and phylum Asgardaeota (1 OTU; 0.39 EAF) were found to assimilate ^13^C-carbon from ^13^C-glucose (“g”; Figs 3 and 5).

We identified 25 OTUs that exhibited a preference for methionine, as their mean ^13^C-carbon assimilation from ^13^C-methionine was higher than their mean ^13^C-carbon assimilation from ^13^C-glucose (Fig. 4A). The strongest methionine-preferring OTUs displayed at least a 2-fold increase in ^13^C-EAF relative to glucose, and were observed with OTUs affiliated with the archaeal genus *Methanococcoides* (1 OTU; 0.26 EAF), assigned and unassigned bacterial genera of *Neptuniibacter*, Gemmatimonadaceae (1 OTU; 0.28 EAF), BD2–11 terrestrial group (1 OTU; 0.33 EAF), Gaiellales (1 OTU; 0.24 EAF), OM190 (1 OTU; 0.22 EAF), and Clostridiales (1 OTU; 0.46 EAF) (Fig. 4A). On the other hand, we identified 11 OTUs that exhibited a preference for taurine, which had higher mean carbon assimilation from taurine compared to glucose (Fig. 4B). Of these, the highest ^13^C-EAF were detected in the OTUs affiliated with unassigned genera of SG8–4 (1 OTU; 0.35 EAF), Gammaproteobacteria Incertae Sedis (1 OTU; 0.33 EAF), *Neptuniibacter*, Nitrincolaceae (2 OTUs; 0.31 ± 0.12 EAF), and *Colwellia* (1 OTU; 0.23 EAF) (Fig. 4A). No archaeal OTUs assimilated taurine, highlighting that bacteria were more important for taurine cycling than archaea.

### Phylogenetic organization

In the Blomberg’s K test, ^13^C-glucose utilization exhibited the highest phylogenetic signal among the incubations (K = 0.684; *P* < .05), followed by ^13^C-taurine utilization (K = 0.299; *P* < .05). The ^13^C-methionine utilization had the lowest phylogenetic signal (K = 0.095, *P* > 0.05), suggesting that methionine utilization was randomly distributed across the tree of microbial taxa utilizing this substrate.

### Metagenomics

In the metagenomic analysis, major changes after 10-days incubations were related to the ATP-binding cassette type transporter (ABC-type) transporter for taurine (*TauABC*) with 2-to-5-fold more enrichment in taurine incubation, S-adenosylmethionine synthase *metK* increasing 1.5-to-2-fold in the methionine incubation, and genes related to glucose uptake (*gtsAB* and *glcEFU*) which were 6-fold more abundant in glucose incubation (Fig. S5).

## Discussion

### Quantifying carbon assimilation from DOS by uncultivated bacteria and archaea

Our study aimed to bridge the knowledge gap regarding the active key microbes responsible for turnover of DOS in marine sediment, an important yet poorly constrained biogeochemical process [23]. In the sediment samples obtained from 493 m water depth on the Puerto Rico continental slope, our analyses showed that nearly half of the microbial communities actively cycled taurine and methionine (Figs 1E, 3, and 4). The qSIP analysis of ^13^C-assimilation indicated that bacteria affiliated with Deltaproteobacteria and Gammaproteobacteria were primarily responsible for uptake of ^13^C-methionine and ^13^C-taurine (Figs 2, 3, and 4). The relative abundance of Gammaproteobacterial OTUs showed a general decrease with depth and mirrored the NO_3_^−^ and O_2_ concentration profiles (Fig. 1B and C). In contrast, the vertical *in situ* profile of deltaproteobacterial OTUs increased with depth as dissolved O_2_ and NO_3_^−^ disappeared (Fig. 1B and C). Deltaproteobacteria are considered to be the dominant group of SRM in global marine sediments [29], playing a significant role in the remineralization of up to 29% of the organic matter deposited to the seafloor [57]. SRM can thrive on diverse substrates such as sugars [58], taurine [59], methionine [60], DMS [22], and methane thiols [61]. Further culture-based studies elucidated that some SRM can utilize several single amino acids including members of *Desulfovibrio* [62], *Desulfobacterium* [63], and *Desulfobulbus* [64]. In line with this finding, we detected a high degree of methionine assimilation by many SRM OTUs of Deltaproteobacteria groups *Desulfobaba*, *Geothermobacter*, *Desulfuromonas*, *Pelobacter*, Sva1033 group, NB1-j group, Desulfuromonadales, Desulfuromonadaceae, Desulfobacteraceae, and Desulfobulbaceae (Figs 3, 4, and S6). All of the ^13^C-labeled deltaproteobacterial genera assimilated more ^13^C from glucose compared to taurine and methionine. One exception to this was a low abundant OTU affiliated with mle1–27 clade (<0.05%) under Deltaproteobacteria (currently under Myxococcota phylum [65]; Fig. 4), a group previously suggested as important putative bacterial predators in wastewater treatment plants [66]. Since this clade is understudied and has been observed in various environments, our qSIP results may suggest either a minor possibility of cross-feeding or metabolic flexibility of its members in marine sediments. These results highlight the mle1–27 clade as a potentially important group for taurine cycling in sediments.

Since the top 2 cmbsf of sediments that was used for the 10-days qSIP incubations contained O_2_, the microbial community structure at the start of the incubations represented a mixture of facultative aerobic to anaerobic microbes (Fig. 1B). In our incubations, the O_2_ concentrations were consumed within hours (Fig. S1), showing that for the overwhelming majority of time the incubations were anoxic. This was supported by species richness analysis and NMDS ordination showing that 10-days incubations had more comparable values to the 2–8 cm sediment communities (anoxic) than the 0–1 cm oxic communities (Figs 1B and S3). This suggests that the establishment of anoxic conditions in the incubations favored the metabolism of facultative anaerobes and to certain extent obligate anaerobes, and that we most likely quantified the anaerobic incorporation of ^13^C-labeled substrates.

In surface marine sediments, sulfide can be rapidly oxidized in the transition zone coupled with chemical oxidants such as Fe and Mn oxides, and microbially mediated sulfide oxidation with NO_3_^−^ [67]. However, our qSIP results pointed toward some facultative anaerobes assimilating the given ^13^C-substrates during the incubations using NO_3_^−^ as terminal electron acceptors. Metagenomic analysis showed that genes involved in NO_3_^−^ reduction and sulfur oxidation (*sox* genes) were most abundant in taurine incubation mostly related with gammaproteobacterial genera of *Amphritea*, *Neptuniibacter*, and *Pontibacterium*, which was consistent with our qSIP results (Fig. S6). Nitrate is a well-known electron acceptor for sulfur-oxidizing bacteria [68] and can also be used as an alternative electron acceptor by some sulfate-reducing bacteria [69]. One facultative anaerobic Gammaproteobacteria, *Alcaligenes* sp. strain NKNTAU, was able to oxidize taurine while using NO_3_^−^ as an electron acceptor [70]. Therefore, it is likely that some of the ^13^C-labeled OTUs affiliated with Gammaproteobacteria in our study might oxidize DOS and glucose using NO_3_^−^ as a terminal electron acceptor. This would explain the substantial increase in relative abundances of Gammaproteobacteria and their relatively higher carbon assimilation rates in taurine incubations (Fig. 1D).

The OTUs taxonomically assigned to putative sulfate-reducing Deltaproteobacteria assimilated more methionine than taurine (Figs 3 and S6), whereas heterotrophic gammaproteobacterial genera showed more ^13^C-labeling with taurine than methionine (Figs 3 and S6). This suggests that although both DOS substrates were utilized by Gammaproteobacteria and Deltaproteobacteria, each group displayed a preference for particular DOS substrates. This difference is reflected in microbial community structure analysis, where taurine addition led to a substantial increase in the relative abundance of OTUs affiliated with Gammaproteobacteria (Fig. 1D). The deltaproteobacterial OTUs generally utilized methionine rapidly compared to other microbial groups. However, they did not show an increase in their relative abundance with the addition of methionine. This might be attributed to slow growth of deltaproteobacterial clades in estuarine [41] and deep-sea sediments [71] or slow metabolism of sulfate reducers [72]. The diversity in sulfur metabolite structures and oxidation states can create opportunities for niche differentiation in marine environments [3]. Therefore, the patterns in DOS substrate preference between Deltaproteobacteria and Gammaproteobacteria might be a result of both genomic capabilities and sulfur oxidation state of the substrates. The more oxidized sulfur-substrate tested here, taurine (sulfonate, S^+4^), appears to have been favored by heterotrophic Gammaproteobacteria. Yet methionine, which is a casamino acid and contains a more reduced form of sulfur (sulfide, S^−2^), was utilized more by putative sulfate-reducing Deltaproteobacteria (Figs 3 and S6). Our findings indicate that the sulfur oxidation states within different DOS molecules may dictate DOS assimilation preferences by specific microbial groups.

### Bacterial taxa exhibiting a preference for DOS

A substantial number of bacterial OTUs exhibited greater methionine catabolism compared to glucose assimilation (Fig. 4A), including the OTUs affiliated with BD2–11 terrestrial group and Gemmatimonadaceae under Gemmatimonadetes phylum. The other members of this phylum were able to grow chemoheterotrophically or photoheterotrophically, using organic substrates such as glucose [73], but their contribution to the sulfur cycle is lacking [73]. The increased carbon assimilation from methionine by multiple bacteria (Fig. 4A), relative to glucose, shows that this DOS substrate is potentially an important carbon source for some benthic microbes. After Deltaproteobacterial genera, three genera affiliated with Firmicutes (*Paramaledivibacter*, unassigned genera of Family XIII and Clostridiales) exhibited high ^13^C-labeling EAFs in methionine incubations, suggesting that Firmicutes are key players in methionine cycling in marine sediments (Figs 3 and 4A). ^13^C-labeled Firmicutes in methionine incubations belonged to the order Clostridiales, which contains spore-forming sulfate-reducers that can also have a significant role in inorganic sulfur cycling and ecosystem functioning as a “seed bank” [74, 75]. Moreover, members of a firmicute, genus *Paramaledivibacter*, have been previously defined as strictly anaerobic microbes which can use methionine as an electron acceptor [76]. In our study, a ^13^C-labeled OTU belonging to genus *Paramaledivibacter* increased its relative abundance from 0.02% in the initial sampling point to 19.5% (Fig. 3), suggesting that this OTU outcompeted the other microbes due to its ability to degrade methionine as a carbon source.

Some bacterial taxa exhibited a higher assimilation of ^13^C from taurine, compared to glucose, indicating that they prefer taurine to glucose as a carbon source (Fig. 4B). Particularly, these were mainly OTUs assigned to the genus *Neptuniibacter* (Family Nitrincolacaea), SG8–4 (Planctomycetes), and the Nitrincolaceae family (Fig. 4B). The SG8–4 group bacteria likely degrade polysaccharides [77], and Nitrincolaceae have the potential for DMSP utilization and methanesulfonate uptake [78], indicating their potential for DOS cycling in marine environments. Of note, a taurine-assimilating OTU belonging to genus *Neptuniibacter* exhibited an increase in relative abundance over several orders of magnitude (Figs 3 and 4). This OTU also assimilated methionine, but not glucose, suggesting that taurine and methionine compounds are important carbon sources for this taxon. *Neptuniibacter* is widespread in marine environments [79] and has been found to degrade taurine [80]. Similarly, OTUs affiliated with the uncultivated group SG8–4 and the members of Nitrincolacaea also assimilated taurine to a larger extent than glucose (Fig. 4B), suggesting that they may prefer DOS as a carbon source over glucose.

### The role of archaea in benthic DOS and glucose cycling

Marine archaea have been found to assimilate taurine in oxic seawater [16], but no benthic archaeal OTUs utilized taurine in our study under anoxic conditions (Fig. 3). On the other hand, three archaeal groups assimilated ^13^C-methionine including the methanogenic *Methanococcoides* [81]. Together with the Deltaproteobacterial genus *Pelobacter*, this archaeon has the genomic capability to degrade ^13^C_2_-choline, an important substrate on methane production [82]. However, to our knowledge, methionine degradation of *Methanococcoides* in anoxic marine sediments was not demonstrated elsewhere. Our study suggests that some *Methanococcoides* contribute to DOS cycling through methionine utilization, potentially linking this to methane production.

Three archaeal OTUs assimilated ^13^C-glucose and were all affiliated with the Asgard archaea superphylum (Figs 3 and 5). One of these OTUs (Lokiarchaeota) was one of the highest ^13^C-enriched OTUs in the glucose incubations out of all microbial taxa (including bacteria) (^13^C-EAF = 0.64, Fig. 3). This glucose-assimilating Lokiarchaeota was identical to a Lokiarchaeota OTU found in coastal sediments from Germany [56] (Fig. 5), and closely related to two mixotrophic Lokiarchaeota OTUs identified in marine sulfidic sediments [54] (Fig. 5). The other Asgard OTUs affiliated with Odinarchaeota also had a relatively high degree of carbon assimilation from glucose (^13^C-EAF = 0.18 and 0.39; Fig. 3). The ^13^C-glucose assimilating OTUs from Odinarchaeota were clustered in different clades from marine sediments (Fig. 5) including those previously reported as exhibiting autotrophic activity (OTU746; Fig. 5) and mixotrophic activity (OTU1430; Fig. 5). This is consistent with Odinarchaeota genomes having an incomplete [83] or complete carbon fixation pathway [84], suggesting a mixotrophic lifestyle. None of the three ^13^C-labeled Asgard OTUs from our study showed ^13^C-DOS assimilation, which is consistent with the lack of genes responsible for the uptake of taurine and methionine in their genomes [56]. These substrate preferences may be relevant for the ecology of Asgard archaea, as pertains to their role in eukaryogenesis theories [85, 86]. For instance, our qSIP results demonstrating a strong preference for glucose by Asgard archaea would suggest that glucose may have been a more desirable metabolite for the primordial proto-mitochondrial symbiont [87] to share with the Asgard host cell, rather than the DOS substrates methionine or taurine.

## Conclusion

Our study bridged a significant knowledge gap regarding the active key microbes responsible for the turnover of important DOS substrates taurine and methionine in marine sediment, an important yet poorly constrained biogeochemical process [23, 29]. Here, we identified for the first time by qSIP the microbial groups that are the principal drivers of active taurine and methionine cycling in anoxic marine sediments. Our results highlight the importance of SRM and Gammaproteobacteria in DOS cycling (Figs 1, 3, and 4). We also observed that globally widespread clades of Gammaproteobacteria and Deltaproteobacteria had distinct preferences for taurine and methionine (Figs 3 and 4), likely due to the differences in their metabolic capabilities. Additionally, our qSIP analysis revealed that taurine utilization was confined to bacteria, whereas no benthic archaea assimilated taurine under anoxia (Fig. 3). Only one OTU from *Neptuniibacter* was found to assimilate taurine and methionine, but not glucose, implying that exclusive microbial specialization on DOS substrates as a carbon source in marine sediments is rare (Figs 3 and 4). Still, a substantial number of bacterial taxa exhibited a higher assimilation ^13^C from taurine or methionine, compared to glucose, indicating their preference for DOS over glucose as a carbon source in the sediment (Fig. 4). Methionine was degraded by bacteria and archaea, including known methanogenic *Methanococcoides* (Fig. 3), raising the possibility of a link between DOS cycling and methane production. The majority of taxa assimilated glucose, supporting heterotrophy as a widespread metabolism in marine sediments [88] (Figs 1E and 3). Among these heterotrophs, three archaeal OTUs of Lokiarchaeota and Odinarchaeota assimilated ^13^C-glucose but not ^13^C-DOS, pointing towards a carbon substrate source different than taurine or methionine in Asgard archaea (Figs 3 and 5). Altogether, our findings provide the first the first taxon-resolved quantitative assessment of DOS incorporation by specific benthic microbial taxa and lays the foundation for identifying key microbial players for DOS cycling in marine environments.

## Supplementary Material

Coskun_ISMEcomm_Supplementary_Information_accepted_ycaf033

Coskun_ISMEcomm_Supplementary_Information_accepted_ycaf033

Coskun_ISMEComm_DataS1_ycaf033

## Data Availability

All data needed to evaluate the paper are present in the main text or the supplementary materials. The intermediate files to replicate the study findings can be found in the Figshare repository under the following link: https://figshare.com/s/84f617866cbd2324e35d. The 16S rRNA gene sequences can be obtained from SRA using the accession numbers between SRR29851295–SRR29851536. The raw 16S rRNA amplicon sequences and metagenomes were entered in the NCBI Sequence Read Archive under BioProject ID PRJNA1136705.
