# Supplementary material for: Identifying the active microbes driving organosulfur cycling from taurine and methionine in marine sediment
Source: ISME Commun. 2025 Feb 25;5(1):ycaf033. doi: 10.1093/ismeco/ycaf033 (PMC11905757; doi:10.1093/ismeco/ycaf033)
Supplement: Coskun_ISMEcomm_Supplementary_Information_accepted_ycaf033 [file coskun_ismecomm_supplementary_information_accepted_ycaf033.pdf]

Supplementary Information

Identifying the active microbes driving organosulfur cycling from taurine and methionine in marine sediment

Ömer K. Coskun[1*], William D. Orsi[1,2], Steven D'Hondt[3], Gonzalo V. Gomez-Saez[1,2*]

[1] Department of Earth and Environmental Sciences, Ludwig-Maximilians-Universität München, 80333 Munich, Germany
[2] GeoBio-Center[LMU], Ludwig-Maximilians-Universität München, 80333 Munich, Germany
[3] Graduate School of Oceanography, University of Rhode Island, 02882 Rhode Island, USA

[*]Corresponding authors:

Gonzalo V. Gomez-Saez, Department of Earth and Environmental Sciences, Ludwig-Maximilians-Universität München, 80333 Munich, Germany. Email: g.gomez@lmu.de

Ömer K. Coskun, Department of Earth and Environmental Sciences, Ludwig-Maximilians-Universität München, 80333 Munich, Germany. Email: o.coskun@lrz.uni-muenchen.de

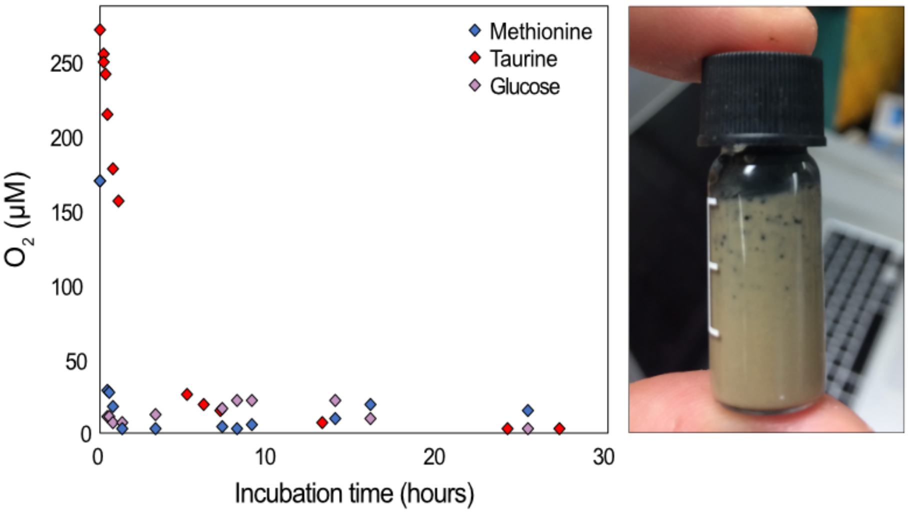

Figure S1. Benthic dissolved O_2 concentrations during the incubations with ^{13}C-labeled substrates.

Photo shows the black precipitates formed in the incubations, likely indicating ongoing sulfate-reduction in the incubation.

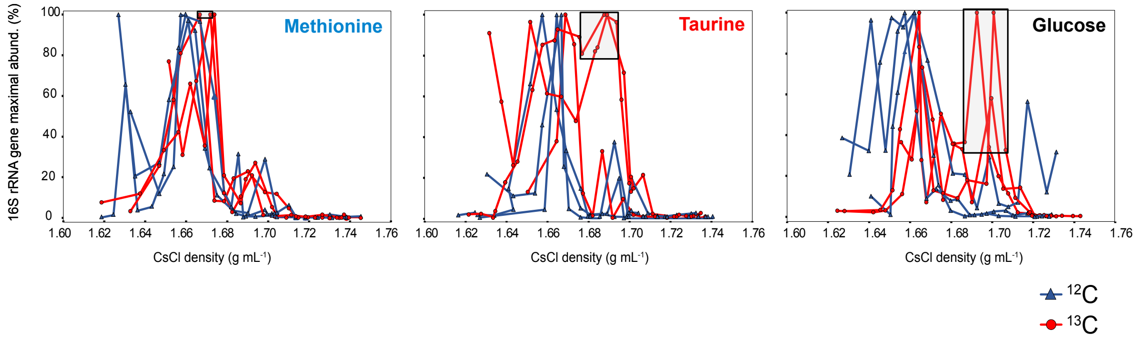

Figure S2. Density gradient shifts between ^{12}C- and ^{13}C-labeled incubations.

Quantification of 16S rRNA gene copies across CsCl density gradient fractions after 10-days of incubation with DOS and glucose substrates. ^{13}C-labeled incubations are represented by colored solid lines with circles and control incubations (^{12}C) are displayed with black dashed lines with triangles.

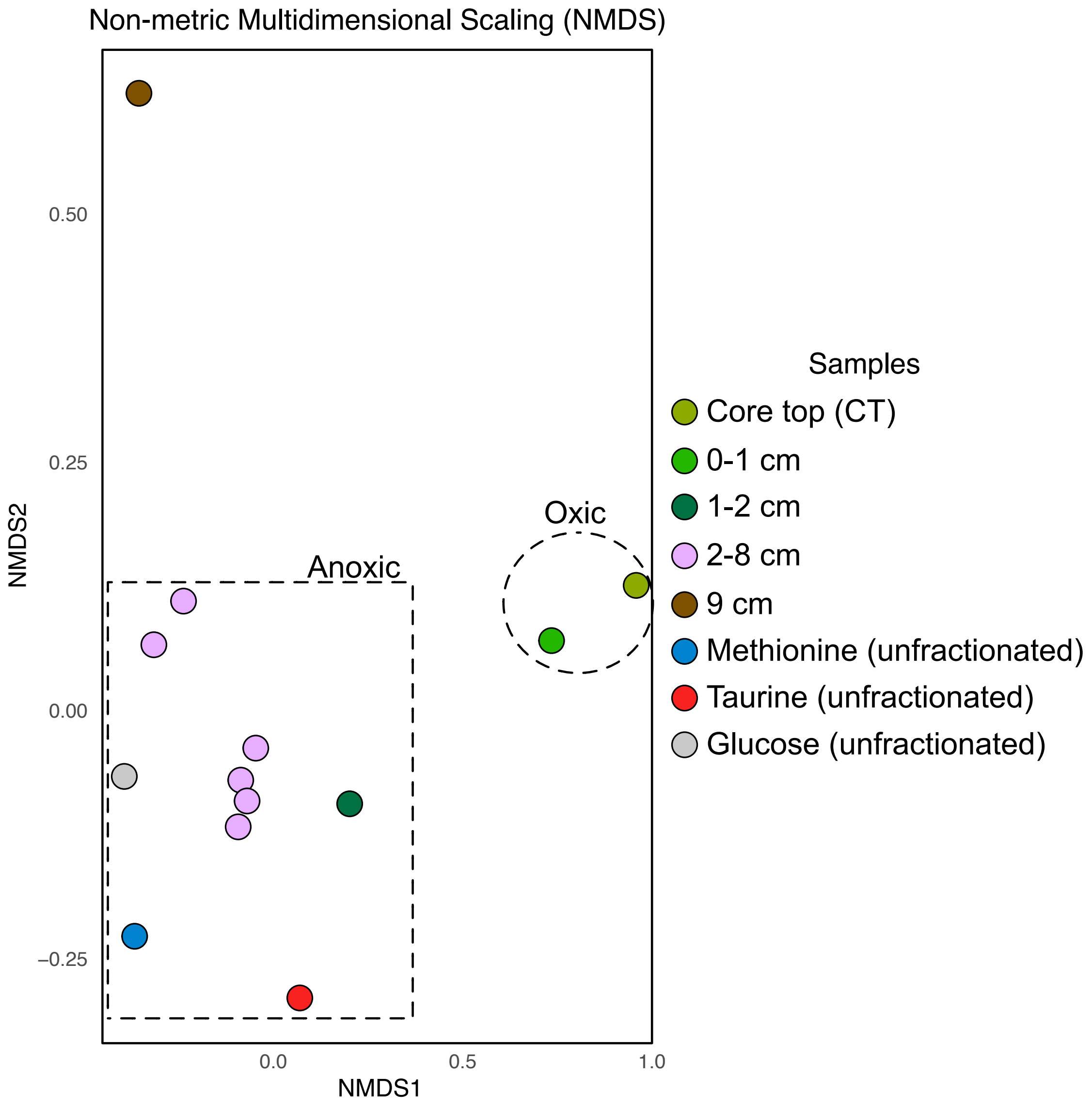

Figure S3. Non-multimetric multidimensional scaling (NMDS) of the in situ and at 10 days microbial communities.

NMDS ordination was achieved using Bray-Curtis dissimilarities distance matrix. Each point represents microbial composition either from in situ sediment profile or from after 10-days of incubation. Note that the microbial composition of unfractionated ^{13}C-labeled incubations at 10-days were more closely associated with the microbial composition obtained from 2-8 cm of sediment, where anoxic conditions prevailed.

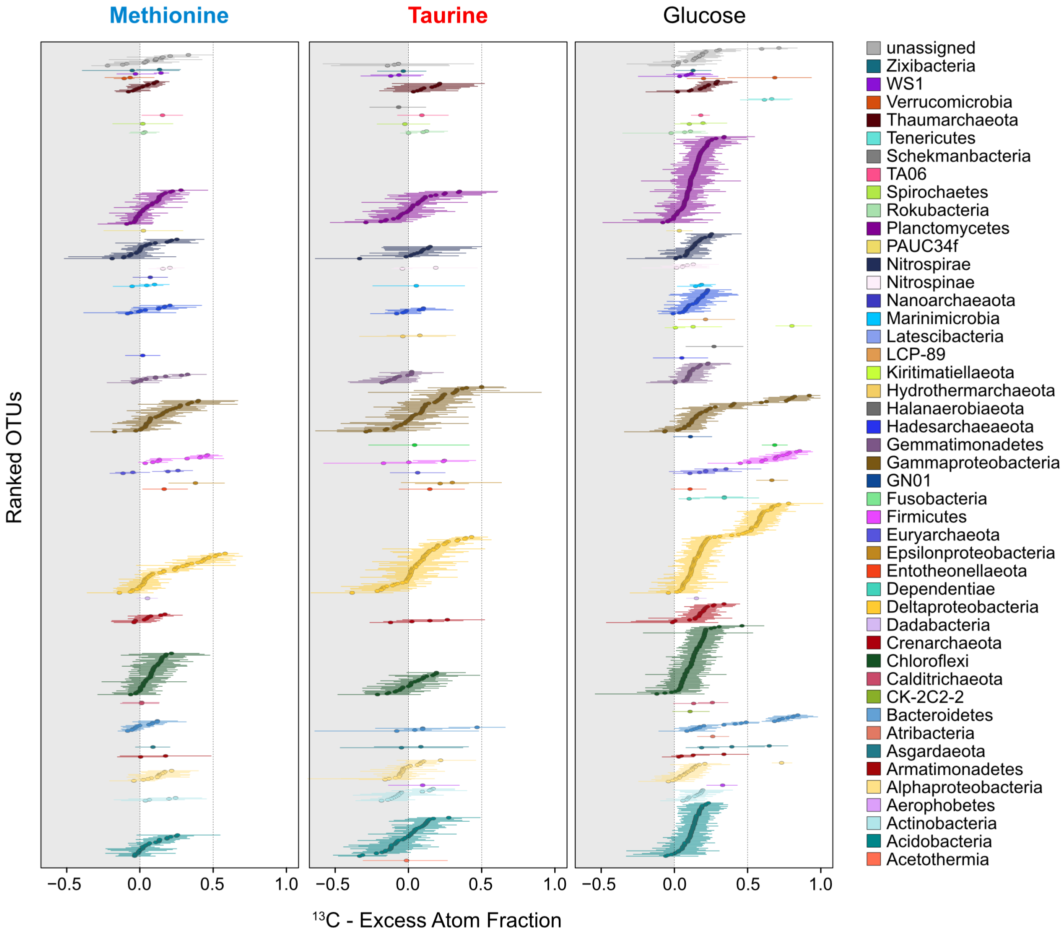

Figure S4. **^{13}C-Excess atom fraction (EAF) in the DNA of OTUs after 10-days incubation.**

Individual points represent median EAF values of specific OTUs across the three replicates. Error bars correspond to 90% confidence intervals across the replicates (e.g., 0.5 indicates 50% of C atoms are ^{13}C-labeled). The “x-axis” is the percent of ^{13}C-labeled carbon atoms in 16S rRNA genes per population. Positive EAF values with confidence intervals not overlapping zero (gray area) are statistically significant. The EAF values for the OTUs are vertically arranged from top to bottom for each major group (the “y-axis”) from the highest EAF value (most ^{13}C-enriched OTU) gradually decreasing to the OTU with the lowest EAF value. For the taxonomic assignment, OTUs are colored by phylum (class in the case of Proteobacteria).

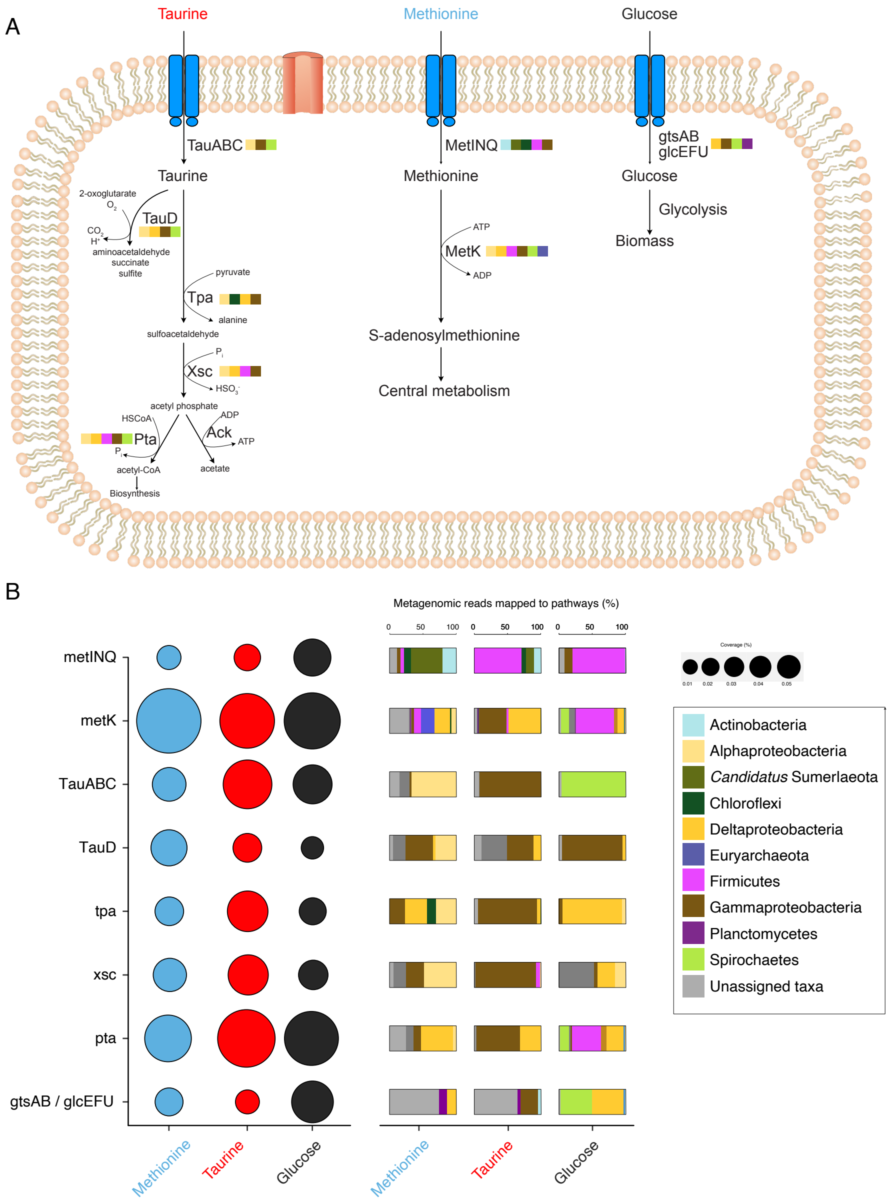

Figure S5: Metabolic potential of microbial groups encoding the genes involved in the utilization of taurine, methionine and glucose.

(A) Open reading frames (ORFs) which were annotated for the utilization of DOS and glucose substrates were selected for the cell diagram. Blue rectangles in the cell membrane represent ATP-binding cassette-type (ABC-type) transporters which are responsible for the uptake or export of the substrates. Taxonomical representation of each gene/gene cluster was shown with rectangles next to them. (B) Left panel represents the relative abundance of the metagenomic reads mapped to each gene/gene cluster whereas right panel shows the taxonomical composition for each gene/gene cluster. *TauABC*: Taurine ABC-type transporters, *TauD*: Alpha-ketoglutarate-dependent taurine dioxygenase, *Tpa*: Taurine:pyruvate aminotransferase, *Xsc*: sulfoacetaldehyde acetyltransferase, *Pta*: Phosphate acetyltransferase, *Ack*: Acetate kinase, *MetINQ*: DL-methionine ABC-type transporter, *MetK*: S-adenosylmethionine synthase, *gtsAB*/*glcEFU*: glucose/mannose ABC-type transport system

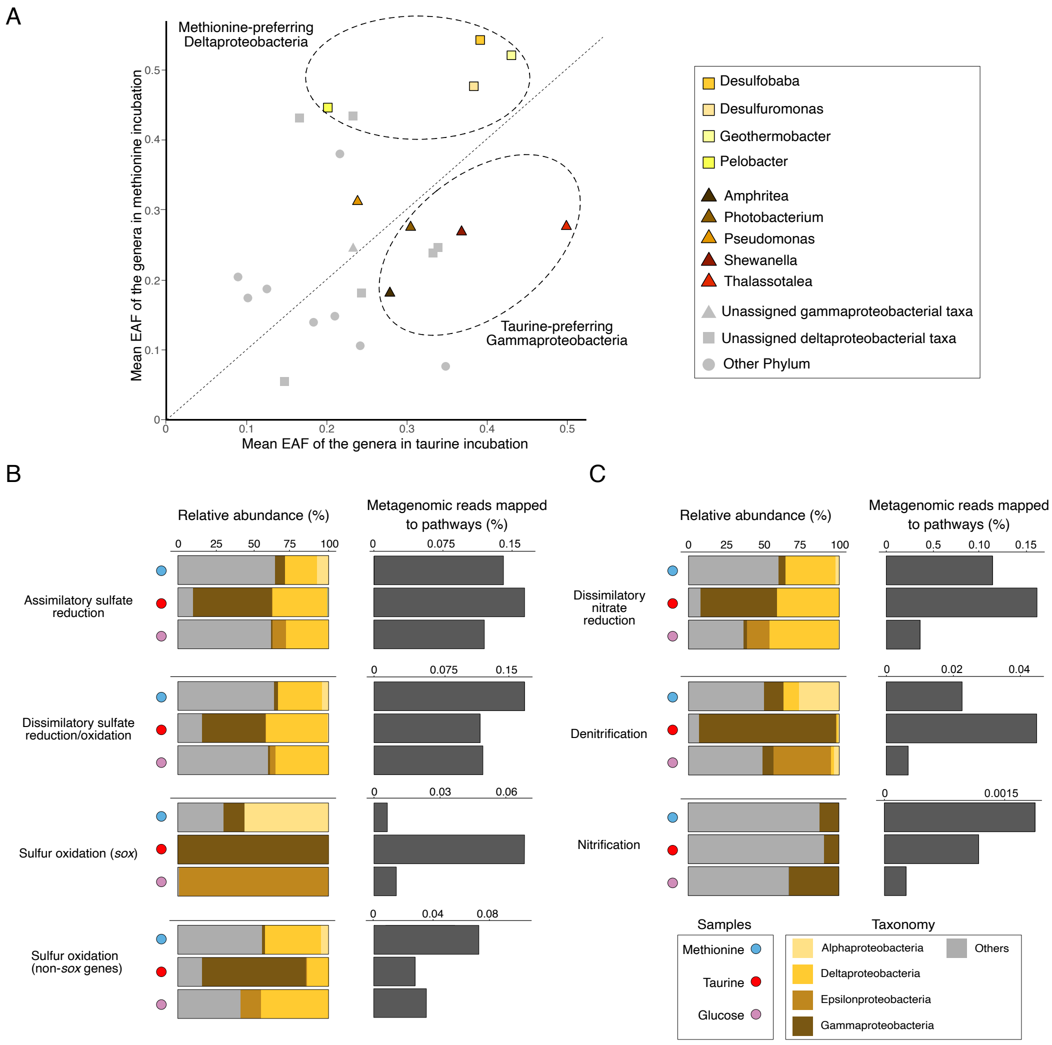

Figure S6. Mean ^{13}C-EAF patterns of ^{13}C-labeled Deltaproteobacteria and Gammaproteobacteria in methionine and taurine incubations and metabolic potential for inorganic sulfur and nitrogen.

(A) ^{13}C-labeled OTUs that exhibited significant labeling across all the incubations were selected for the plot, which corresponds to section "a" of Figure 3. Each point represents the mean EAFs of the OTUs at genus level. For genera with more than one OTU, each point represents the mean EAFs calculated from the average of bootstrapped median EAF of those corresponding OTUs. The ^{13}C-labeled OTUs that were not assigned to a genus in Deltaproteobacteria and Gammaproteobacteria are represented as "Unassigned genus". Squares with the shades of yellow and orange are used for deltaproteobacterial genera, and triangles ranging from brown to red are used for gammaproteobacterial genera. (B-C) The stacked bar plots represent the taxonomic bar charts for the corresponding metabolic pathway related to inorganic sulfur cycle (B), inorganic nitrogen cycle (C) at the phylum level in the "heavy fraction" metagenomes of methionine, taurine and glucose. The dark-gray colored bar plots next to taxonomic charts in each panel correspond the relative abundance (% reads mapping, length normalized) within the different metagenomes. ORFs with best BLASTp similarity to the functional genes in inorganic sulfur, inorganic nitrogen and ABC-type transporters taking part in taurine, methionine and branched amino acids were selected for plot generation.

Supplementary Data S1: qSIP results of the incubations amended with taurine, methionine and glucose.

Please find Data S1 as an attached excel file to the Supplementary Material.
